# Lewy-related pathology exhibits two anatomically and genetically distinct progression patterns: a population-based study of Finns aged 85+

**DOI:** 10.1007/s00401-019-02071-3

**Published:** 2019-09-07

**Authors:** Anna Raunio, Karri Kaivola, Jarno Tuimala, Mia Kero, Minna Oinas, Tuomo Polvikoski, Anders Paetau, Pentti J. Tienari, Liisa Myllykangas

**Affiliations:** 1 Department of Pathology, HUSLAB, Helsinki University Hospital, University of Helsinki, P.O. Box 21, 00014 Helsinki, Finland; 2 Translational Immunology, Research Programs Unit, University of Helsinki, Helsinki, Finland; 3 Department of Neurology, Helsinki University Hospital, P.O. Box 63, 00014 Helsinki, Finland; 4 Department of Pathology, University of Helsinki, P.O. Box 21, 00014 Helsinki, Finland; 5 Department of Neurosurgery, Helsinki University Hospital, University of Helsinki, P.O. Box 21, 00014 Helsinki, Finland; 6 Institute of Neuroscience, Newcastle University, Newcastle upon Tyne, NE2 4HH UK

**Keywords:** Lewy-related pathology, α-Synuclein, Population-based, Aged, 80 and over, Lewy body diseases, Alzheimer’s disease

## Abstract

According to a generally accepted concept Lewy-related pathology (LRP) follows hierarchical caudo-rostral progression. LRP is also frequently present concomitantly with Alzheimer’s disease (AD), and it has been hypothesized that AD-associated LRP forms a distinct type of α-synucleinopathy, where LRP originates in the amygdala. The frequency of distinct forms of LRP progression types has not been studied in a population-based setting. We investigated the distribution and progression of LRP and its relation to AD pathology and *apolipoprotein* (*APOE)* ε4 in a population-based sample of Finns aged over 85 years (*N* = 304). Samples from spinal cord to neocortical areas representing 11 anatomical sites without any hierarchical selection were analyzed immunohistochemically (α-synuclein antibody clone 5G4). LRP was present in 124 individuals (41%) and according to DLB Consortium guidelines 19 of them were categorized as brainstem, 10 amygdala-predominant, 41 limbic, and 43 diffuse neocortical type, whereas 11 could not be classified. To determine the LRP progression patterns, a systematic anatomical scoring was carried out by taking into account the densities of the semiquantitative LRP scores in each anatomic site. With this scoring 123 (99%) subjects could be classified into two progression pattern types: 67% showed caudo-rostral and 32% amygdala-based progression. The unsupervised statistical *K*-means cluster analysis was used as a supplementary test and supported the presence of two progression patterns and had a 90% overall concordance with the systematic anatomical scoring method. Severe Braak NFT stage, high CERAD score and *APOE* ε4 were significantly (all *p* < 0.00001) associated with amygdala-based, but not with caudo-rostral progression type (all *p* > 0.2). This population-based study demonstrates two distinct common LRP progression patterns in the very elderly population. The amygdala-based pattern was associated with *APOE* ε4 and AD pathology. The results confirm the previous progression hypotheses but also widen the concept of the AD-associated LRP.

**Electronic supplementary material:**

The online version of this article (10.1007/s00401-019-02071-3) contains supplementary material, which is available to authorized users.

## Introduction

Dementia with Lewy bodies (DLB) is considered the second most common primary neurodegenerative disease after Alzheimer’s disease (AD) [44, 45]. Its pathological hallmark is Lewy-related pathology (LRP) consisting of α-synuclein-positive Lewy bodies and neurites [14]. The widely used DLB Consortium guidelines [28] classify LRP into three types based on the extent of LRP in the brain—brainstem, limbic or neocortex—following a generally accepted concept, suggesting that LRP evolves hierarchically, starting in the dorsal medulla oblongata and progressing through brainstem to supratentorial structures (caudo-rostral progression) [1, 6, 23, 28, 29].

LRP is also frequently present in combination with other neurodegenerative diseases, especially AD [38]. The LRP pathology in AD is often most severe or even restricted to amygdala [2, 11, 18, 43], indicating a deviation from the caudo-rostral progression. Due to this, an amygdala-predominant type of LRP was added in the newest DLB Consortium guidelines [27]. It has been proposed that AD-associated LRP forms a distinct type of α-synucleinopathy, in which LRP arises de novo in the amygdala and then progresses to entorhinal cortex, brainstem or both [11, 43]. Recent studies, based on a data-driven cluster analysis, supported this hypothesis [34, 41] and an idea of distinct types of LRP progression has also been raised by some other studies [3, 13]. It is noteworthy, however, that the hypothesis of LRP based in the amygdala is grounded on studies on patients from referral-based institutions, and thus these studies may involve selection bias. Population- or community-based studies that could ascertain the prevalence of the amygdala-based progression pattern in the general population do not exist.

In this study, we determined LRP in 11 central nervous system (CNS) regions in a population-based sample of 304 individuals aged 85 and older (Vantaa 85+). We report that in the very elderly population LRP shows two common progression patterns: (1) the caudo-rostral pattern and (2) the AD-associated amygdala-based pattern with origin in the amygdala.

## Materials and methods

### Study sample

We evaluated the neuropathologically examined subsample of the population-based Vantaa 85+ study. The Vantaa 85+ study consists of all individuals aged at least 85 years who had lived in the city of Vantaa (Southern Finland) on April 1, 1991. Of the 601 eligible individuals 11 refused to participate and one could not be reached. During a 10-year follow-up, 565 died and 304 (54%) were autopsied constituting the neuropathological subsample. The demographic details of the neuropathologically studied subsample have been previously reported [33] and do not deviate from the whole study sample (Supplemental Table 1). The neuropathological subsample consisted of 52 men and 252 women. Age at death ranged from 85 to 105 years. 196 of the deceased had been with dementia and 108 without. The demographic, neuropathologic and genetic details of the subsample with and without dementia are summoned in Table 1.

**Table 1:** Characteristics of the neuropathological subsample, with and without dementia, of the Vantaa 85+ study

	All participants (*n* = 304)	Dementia (*n* = 196)	No dementia (*n* = 108)
Demographical details			
Sex (*n*, %)			
Men	52 (17)	30 (15)	22 (20)
Women	252 (83)	166 (85)	86 (80)
Age at death (mean ± SD)	92.4 (± 3.7)	92.5 (± 3.7)	92.1 (± 3.8)
Age at death (*n*, %)			
85–89	82 (27)	46 (24)	36 (33)
90–94	146 (48)	101 (52)	45 (42)
≥ 95	76 (25)	49 (25)	27 (25)
Neuropathological details			
Braak NFT stage (*n*, %)			
0–II	90 (30)	46 (24)	44 (41)
III–IV	142 (47)	84 (43)	58 (54)
V–VI	72 (24)	66 (34)	6 (6)
CERAD score (*n*, %)			
None	71 (23)	33 (17)	38 (35)
Sparse	33 (11)	17 (9)	16 (15)
Moderate–frequent	200 (66)	146 (75)	54 (50)
SN neuron loss (*n*, %)^a^			
None	7 (2)	3 (2)	4 (4)
Mild	161 (53)	98 (50)	63 (58)
Moderate	115 (38)	76 (39)	39 (36)
Severe	20 (7)	19 (10)	1 (1)
Genetic characteristics			
*APOE* ε4^b^ (*n*, %)			
No	194 (68)	112 (61)	82 (83)
Yes	90 (32)	73 (39)	17 (17)

*NFT* neurofibrillary tangle, *SN* substantia nigra

^a^SN sample was missing from one participant

^b^DNA samples of 20 participants were not available (*n* = 284)

### Neuropathological procedures

We assessed α-synuclein pathology using immunohistochemistry with mouse monoclonal anti-α-synuclein antibody (clone 5G4, 1:1000, AJ Roboscreen GmbH, Leipzig, Germany or Merck KGaA, Darmstadt, Germany) [25]. We stained 5 μm tissue sections obtained from 11 anatomic sites (sacral and thoracic spinal cord, medulla oblongata, pons, midbrain, amygdala, hippocampus from right hemisphere, cingulate cortex, frontal cortex, temporal cortex and parietal cortex) per individual as previously described [39].

Samples included in this study follow recommendations of the DLB Consortium guidelines [27, 28] apart from the olfactory bulb and nucleus basalis of Meynert, which were not included in the original post-mortem sampling protocol of the Vantaa 85+ study. On the other hand, regions of spinal cord, not belonging to the DLB guidelines, were included in this study and were scored following previously published work, in which spinal cord regions were scored equivalently to brain regions described in the DLB Consortium guidelines [3, 28].

The detailed description of AD pathology of the Vantaa 85+ study has been reported previously [32, 36, 37]. Briefly, the neuritic plaques were assessed by Bielshowsky silver stain according to the CERAD protocol [30] and neurofibrillary tangles by Gallyas silver stain method according to the protocol by Braak and Braak [5]. The neuropathological AD diagnosis was determined according to modified National Institute on Aging (NIA)/Reagan Institute Consensus Recommendations for the Postmortem Diagnosis of AD (NIA-RI) [19]. The neuropathological AD required “moderate” or “frequent” plaque scores according to the CERAD protocol [30] and Braak NFT stages IV–VI of neurofibrillary pathology [5]. The controls were selected from the neuropathologically examined subpopulation and criteria required neuritic plaque score “none” or “sparse” and Braak NFT stage less than III. In addition, Lewy neurites in hippocampal CA2-3 were semiquantitatively assessed as previously recommended [10, 33]. The assessment of the dementia status and analysis of the substantia nigra neuronal loss have been previously reported [33].

### LRP scoring and classification

First, we scored LRP on a semiquantitative scale (0 = none, 1 = mild, 2 = moderate, 3 = severe, 4 = very severe) in the spinal cord (sacral posterior root entry, sacral anterior horn, central canal adjacent to sacral spinal cord and thoracic intermediolateral horn of the thoracic spinal cord), medulla (dorsal nucleus of vagus), pons (locus coeruleus), midbrain (substantia nigra), basal forebrain (amygdala), hippocampus (CA2 and transentorhinal cortex), gyrus cinguli, temporal cortex, frontal cortex and parietal cortex (Supplemental Table 2). We classified individuals into LRP classification types presented in the newest DLB guidelines (brainstem, amygdala-predominant, limbic and diffuse neocortical) [27]. Individuals with LRP in the amygdala and paucity of LRP in other brain regions were classified in the amygdala-predominant type [1, 27, 39]. Individuals that had LRP incomparable to any LRP type even after minor modifications (see Supplemental material for detailed description) to the DLB guidelines were regarded as non-classifiable.

Then, based on LRP scores and LRP classification types, we studied if individuals could fit consistently to the previously hypothesized caudo-rostral and amygdala-based progression patterns. Thus, the systematic anatomical scoring, taking into account differences in density and distribution profiles of the semiquantitative LRP scores, was applied. Our guidelines for categorizing LRP progression pattern according to the systematic anatomical scoring were in brief:

(A) Caudo-rostral pattern was defined by the strongest LRP at the medulla and brainstem area, from where the pathology spreads through limbic areas to cerebral neocortex as the disease progresses.
(B) Amygdala-based pattern was defined by the strongest pathology in the amygdala or in limbic area and propagation to both caudal (brainstem, medulla and spinal cord) and rostral neocortical regions.
(C) One subject could not be categorized as every brain region was scored as very severe.

### Statistical analyses

The stability of the LRP scored by the systematic anatomical scoring was then assessed using an unsupervised *K*-means classification method [22]. Although *K*-means does not enable direct validation of the results, it gives visual cues for the hard-to-classify samples, and aids analysts in their classification efforts. Association between variables, such as LRP progression pattern, Braak NFT stage, CERAD score, *APOE* ε4, and gender was assessed using Fisher’s exact test. Means of continuous variables between two groups were compared using *t* test. If there were several groups to compare, a linear regression model was used. All statistical analyses were performed in R version 3.6.0 (R Core Team 2019 R: A language and environment for statistical computing. R Foundation for Statistical Computing, Vienna, Austria. URL https://www.R-project.org/).

### Ethics

The Vantaa 85+ study was approved by the Ethics Committee of the Health Centre of the City of Vantaa, and by the Coordinating Ethics Committee of the Helsinki and Uusimaa Hospital District. The Finnish Health and Social Ministry permitted the use of health and social work records and death certificates. The National Authority for Medicolegal Affairs (VALVIRA) approved the collection of the tissue samples and their use for research. The study participants or their relatives gave informed consent for blood sample collections and clinical evaluations. A written consent for each autopsy was obtained from the next of kin [37].

## Results

**Fig. 1:**
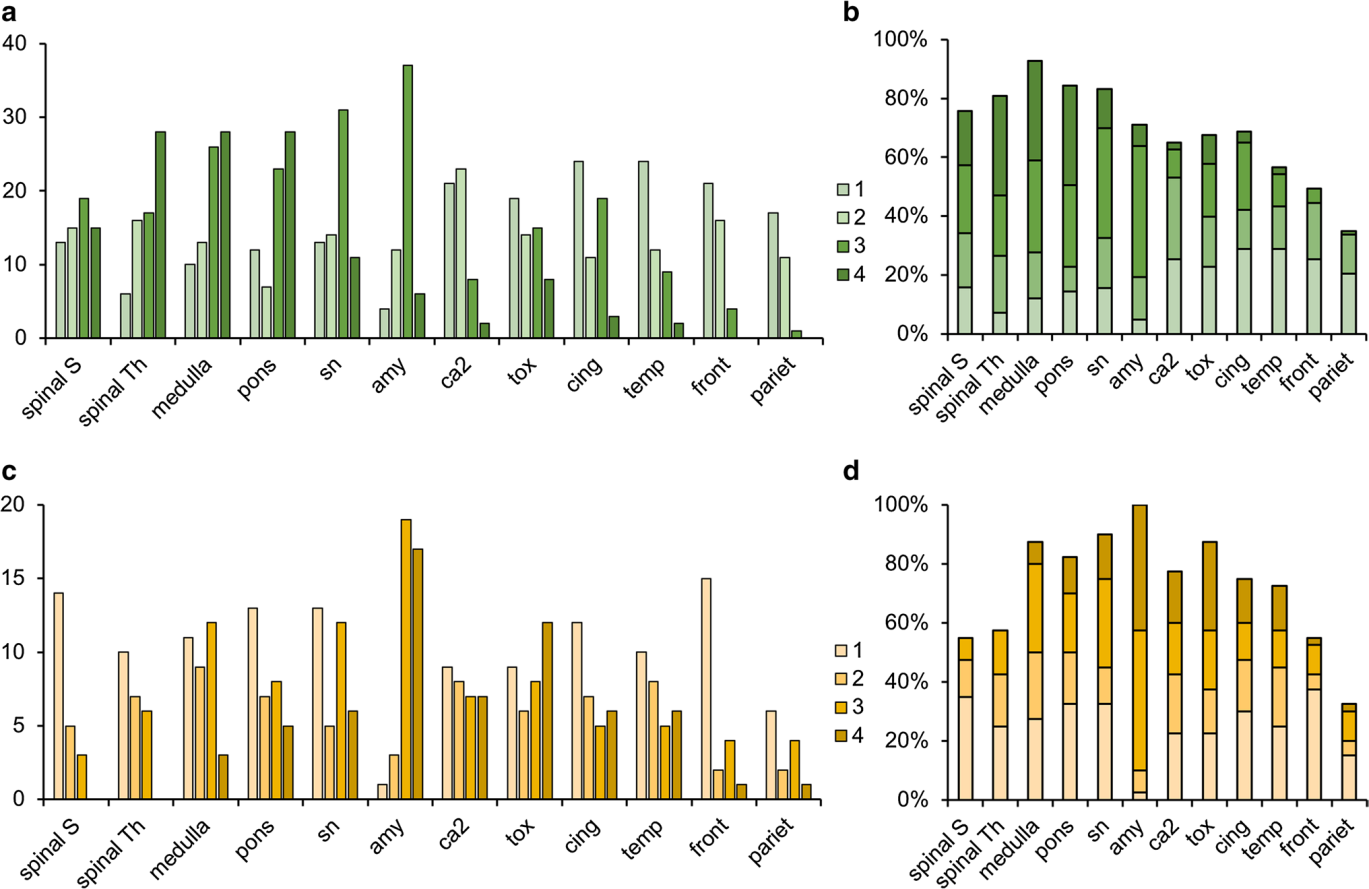
Distribution and density of Lewy-related pathology in the investigated brain regions. **a**, **b** Caudo-rostral *n* = 83 and **c**, **d** amygdala-based *n* = 40 progression patterns visualized (*y*-axis) by quantity (*n*) and percentage (%). Mean values (SD) of the investigated brain regions are shown in Supplemental Table 4. Spinal S = sacral spinal cord, spinal Th = thoracic spinal cord, sn = substantia nigra, amy = amygdala, ca2 = ca2 of hippocampus, tox = transentorhinal cortex of hippocampus, cing = cingulate cortex, temp = temporal cortex, front = frontal cortex, pariet = parietal cortex, 1 = mild, 2 = moderate, 3 = severe, 4 = very severe Lewy-related pathology

Of the neuropathologically examined subsample (*n* = 304), LRP was present in at least one of the scored areas in 124 subjects (41%), while 180 individuals had no LRP. When present, LRP was most frequently observed in the medulla (*n* = 113) and the substantia nigra (*n* = 106), and most infrequently in the parietal cortex region (*n* = 43) (Fig. 1).

### DLB Consortium classification

After minor adjustments to the DLB Consortium guidelines (see Supplemental material for detailed description), we could classify 113/124 (91%) individuals into LRP classification types; 19 were brainstem, 10 amygdala-predominant, 41 limbic, and 43 diffuse neocortical type (Table 2). One subject showed, in addition to limbic type of α-synuclein pathology, strong oligodendroglial α-synuclein pathology compatible with multiple system atrophy. 11 (9%) individuals could not be classified into DLB Consortium types. They showed LRP confined in the pons, medulla and/or spinal cord, and no LRP was found in other brain regions. In addition, their neuron loss score in the substantia nigra was at most mild (Table 3).

**Table 2:** The LRP progression patterns compared to the DLB Consortium classification [27] of the neuropathological subsample of the Vantaa 85+ Study (*n* = 304^a^)

	DLB Consortium classification					
	None *n* = 180	Non-classifiable *n* = 11	Brainstem *n* = 19	Amygdala-predominant *n* = 10	Limbic *n* = 41	Diffuse neocortical *n* = 43
LRP progression patterns^b^						
None *n* = 180 (59%)	180 (100)					
Caudo-rostral pattern *n* = 83 (27%)		11 (100)	18 (95)	0 (0)	29 (71)	25 (58)
Amygdala-based pattern *n* = 40 (13%)		0 (0)	1 (5)	10 (100)	12 (29)	17 (40)

*LRP* Lewy-related pathology, *DLB* dementia with Lewy bodies

^a^Hippocampal samples from the right hemisphere were missing from 2 participants and were substituted with the left hemisphere samples; both spinal cord samples were missing from 1 participant, sacral spinal cord sample from 1 participant and SN sample from 1 participant

^b^One subject had highest LRP stage in all brain regions and the progression pattern could not be determined

**Table 3:** Characteristics of the neuropathological subsample of the Vantaa 85+ Study *n* = 304^a^ categorized by (a) DLB Consortium classification [27], (b) LRP progression-based classification

	Negative LRP	Positive LRP (Lewy-related pathology) *n* = 124						
		(a) DLB Consortium classification [27] *n* = 124					(b) LRP progression-based *n* = 123^b^	
	No *n* = 180	Non-classifiable *n* = 11	Brainstem *n* = 19	Amygdala-predominant *n* = 10	Limbic *n* = 41	Diffuse Neocortical *n* = 43	Caudo-rostral *n* = 83	Amygdala-based *n* = 40
Women (%)	85	82	84	90	80	74	76	88
Mean age at death (years)	92.3	91.3	93.8	93.0	92.3	92.2	92.6	92.3
Age at death (*n*, %)								
85–89	45 (25)	5 (46)	4 (21)	1 (10)	14 (34)	13 (30)	26 (31)	11 (28)
90–94	94 (52)	3 (27)	9 (47)	6 (60)	16 (39)	18 (42)	33 (40)	18 (45)
≥ 95	41 (23)	3 (27)	6 (32)	3 (30)	11 (27)	12 (28)	24 (29)	11 (28)
Braak NFT stage (*n*, %)								
0-II	54 (30)	6 (55)	8 (42)	1 (10)	10 (24)	11 (26)	34 (41)	2 (5)
III–IV	92 (51)	3 (27)	8 (42)	5 (50)	20 (49)	14 (33)	36 (43)	13 (33)
V–VI	34 (19)	2 (18)	3 (16)	4 (40)	11 (27)	18 (42)	13 (16)	25 (63)
CERAD score (*n*, %)								
None	46 (26)	2 (18)	7 (37)	1 (10)	11 (27)	4 (9)	24 (29)	1 (3)
Sparse	24 (13)	1 (9)	3 (16)	0 (0)	3 (7)	2 (5)	9 (11)	0 (0)
Moderate–frequent	110 (61)	8 (73)	9 (47)	9 (90)	27 (66)	37 (86)	50 (60)	39 (98)
NIA-RI^c^ (*n*, %)								
No	32 (35)	3 (50)	7 (58)	0 (0)	7 (26)	4 (13)	21 (25)	0 (0)
Yes	59 (65)	3 (50)	5 (42)	7 (100)	20 (74)	26 (87)	24 (29)	36 (90)
SN neuron loss^d^ (*n*, %)								
None	6 (3)	0 (0)	0 (0)	0 (0)	1 (2)	0 (0)	0 (0)	1 (3)
Mild	115 (64)	11 (100)	9 (47)	5 (50)	16 (39)	5 (12)	36 (43)	10 (25)
Moderate	54 (30)	0 (0)	8 (42)	5 (50)	20 (49)	28 (65)	36 (43)	25 (63)
Severe	4 (1)	0 (0)	2 (11)	0 (0)	4 (10)	10 (23)	11 (13)	4 (10)
Dementia status at death (*n*, %)								
No	74 (41)	5 (45)	10 (53)	1 (10)	15 (37)	3 (7)	31 (37)	3 (8)
Yes	106 (59)	6 (55)	9 (47)	9 (90)	26 (63)	40 (93)	52 (63)	37 (93)
Age at dementia onset^e^	87.2	86.5	88.5	88.1	88.4	86.0	88.5	85.3
Duration of dementia	5.2	5.6	4.8	5.3	4.3	6.1	4.2	6.9
*APOE* ε4^f^ (*n*, %)								
No	126 (74)	7 (70)	15 (79)	5 (56)	19 (53)	22 (55)	54 (65)	13 (33)
Yes	44 (26)	3 (30)	4 (21)	4 (44)	17 (47)	18 (45)	24 (29)	22 (55)

Severe AD pathology (Braak NFT and CERAD score) and *APOE* ε4 are significantly more common in subjects with the amygdala-based progression pattern compared to those with caudo-rostral pattern or individuals with no LRP

*LRP* Lewy-related pathology, *DLB* dementia with Lewy bodies, *NFT* neurofibrillary tangles, *SN* substantia nigra

^a^Hippocampal samples from the right hemisphere were missing from 2 participants and were substituted with the left hemisphere samples; both spinal cord samples were missing from 1 participant, sacral spinal cord sample from 1 participant and SN sample from 1 participant

^b^One subject could not be classified because all regions obtained highest score and was excluded

^c^Modified NIA-RI neuropathological AD defined by [36] *n* = 173

^d^Excluded 1 participant without SN sample

^e^missing age at onset and duration of dementia values from 3 participants

^f^DNA samples of 20 participants were not available, *n* = 284

### LRP progression patterns

Based on LRP scores and LRP classification types, we next studied if individuals could fit consistently to the previously hypothesized caudo-rostral and amygdala-based progression patterns by systematic anatomical scoring. From the 124 individuals with LRP, 83 (67%) showed caudo-rostral progression and 40 (32%) showed amygdala-based progression. In the whole population 27% showed LRP with caudo-rostral and 13% with amygdala-based progression pattern. One individual had the highest LRP stage 4 in all brain regions and the progression pattern could not be determined (Table 3).

**Fig. 2:**
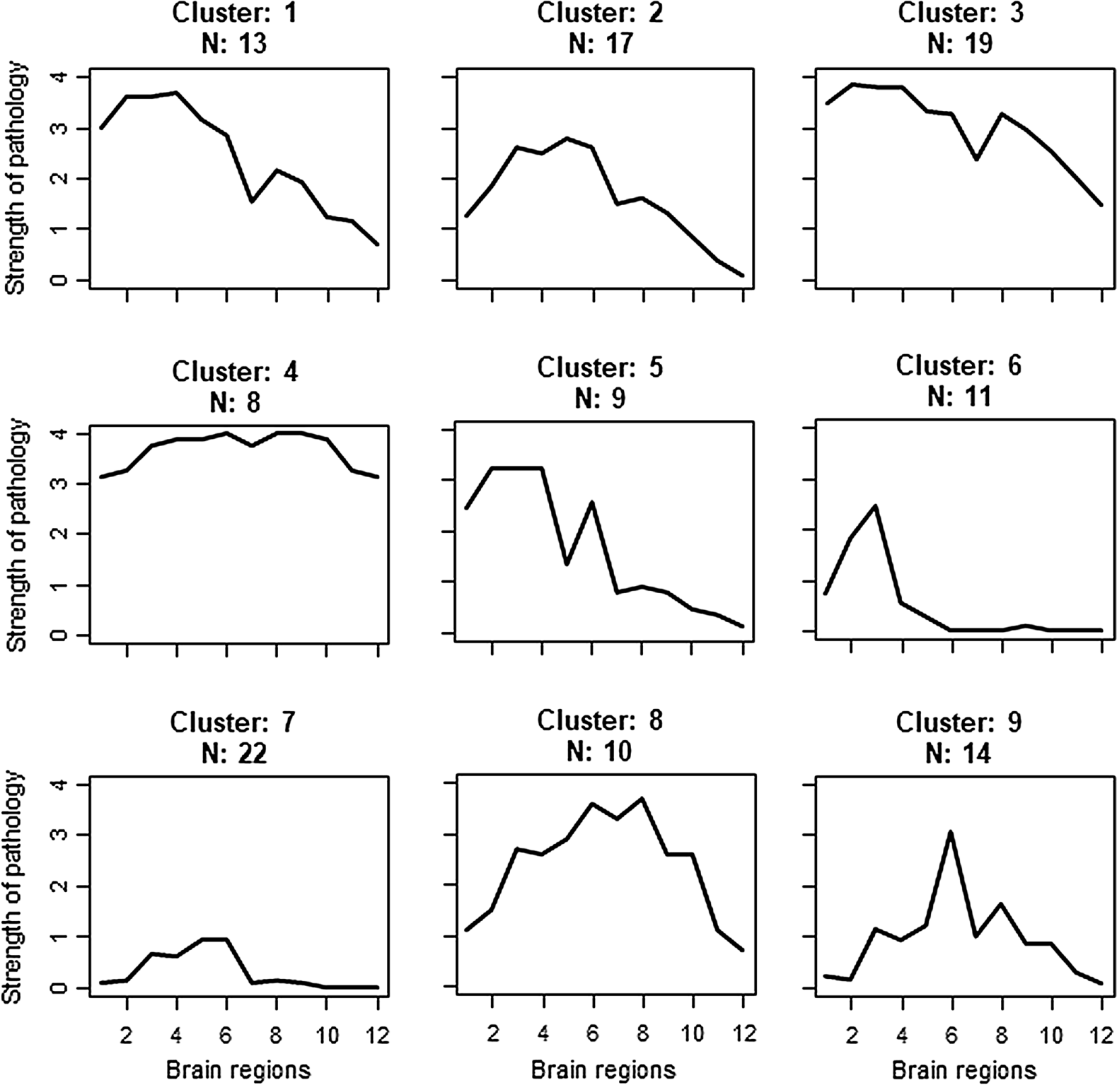
Classification of individuals by the progression pattern of LRP by *K*-means cluster analysis. On the *y*-axis is the semiquantitative LRP score (0–4). On the x-axis are the different CNS regions from spinal cord to neocortex: 1 = sacral spinal cord, 2 = thoracic spinal cord, 3 = medulla, 4 = pons, 5 = substantia nigra, 6 = amygdala, 7 = ca2 of hippocampus, 8 = transentorhinal cortex of hippocampus, 9 = cingulate cortex, 10 = temporal cortex, 11 = frontal cortex, 12 = parietal cortex. Clusters 1, 2, 3, 5 and 6 include individuals with caudo-rostral LRP progression pattern. Clusters 7, 8 and 9 include individuals with amygdala-based progression pattern Cluster 4 includes most severe LRP progression pattern from both caudo-rostral and amygdala-based patterns

*K*-means cluster analysis classified individuals into nine clusters that matched the hypothesis of caudo-rostral and amygdala-based LRP progression. The result with nine clusters was judged optimal using the “elbow method” [22]. Five clusters (*n* = 69) showed the strongest LRP at the spinal and brainstem level with decreasing LRP towards the limbic and cortical areas corresponding to the caudo-rostral LRP progression. Three clusters (*n* = 46) had the strongest LRP in the amygdala or limbic areas with decreasing pathology towards both the spinal-brainstem and cortical regions corresponding to the amygdala-based progression pattern. One cluster, formed by eight individuals, could not be classified (Fig. 2).

Overall, the *K*-means cluster analysis and the systematic anatomical scoring classified 90% into same groups, showing marked concordance between these analyses. Systematic anatomical scoring was used as the final results.

### Overlap of the DLB consortium classification and LRP progression

The overlap of the DLB consortium LRP types and LRP progression patterns is shown in Table 2. Nearly all individuals (95%) of the brainstem type had the caudo-rostral progression pattern, and all of the amygdala-predominant type had the amygdala-based progression pattern. 58% of individuals with the diffuse neocortical and 71% of the subjects with limbic LRP type had the caudo-rostral progression pattern, but both types also had a relatively high proportion (40% and 29%) of subjects with the amygdala-based progression pattern.

### Spinal cord pathology and progression patterns

The distributions of spinal LRP are shown in Fig. 1. As expected, spinal cord pathology was predominantly associated with caudo-rostral progression pattern (Fisher’s test *p* = 0.007219). Out of 83 subjects with caudo-rostral progression pattern, 69 (83%) had detectable spinal LRP, and severe or very severe spinal LRP was present in 45 subjects (54%). Out of 40 subjects with amygdala-based progression pattern, 24 (60%) had any spinal LRP, but severe or very severe spinal LRP (classes 3 or 4) was present in only 6 subjects (15%).

### Demographic characteristics, dementia, and LRP progression patterns

There were slightly more men in the caudo-rostral progression pattern group compared to the amygdala-based progression pattern group (24% vs. 12%), but the difference was not statistically significant (Fisher’s test, *p* = 0.1576). The mean age at death did not show significant differences (*p* = 0.7256) between the amygdala-based (92.3 years), caudo-rostral (92.6 years) or no LRP (92.3 years) groups (Table 3).

There were significantly more people with dementia in the amygdala-based progression pattern group than in the caudo-rostral group (Fisher’s test, *p* = 0.0004478) or in individuals with no LRP (Fisher’s test, *p* = 0.000018). The mean age of onset of dementia was 3.3 years lower in the amygdala-based than in the caudo-rostral progression pattern group (85.3 vs. 88.5 years, *p* = 0.003192), but the average survival time after the onset of dementia was almost 3 years longer in the amygdala-based pattern group (*p* = 0.0007487). Similar trend was seen when subjects with amygdala-based progression pattern were compared with individuals with no LRP (age at onset of dementia, *p* = 0.05216; duration of dementia, *p* = 0.01992). When comparing the caudo-rostral pattern group with individuals without LRP, there were no significant differences in the duration of dementia (*p* = 0.09785) or at the age at onset of dementia (*p* = 0.06853).

### AD pathology and LRP progression patterns

The amygdala-based progression pattern was strongly associated with AD pathology (data shown in Table 3). Severe Braak NFT stage (V–VI) was more common in individuals with the amygdala-based progression than in those with the caudo-rostral progression (Fisher’s test, *p* value 0.00000005632) or with no LRP (Fisher’s test, *p* value = 0.0000001051). High CERAD score (moderate to frequent) was also more common in individuals with the amygdala-based progression than in those with the caudo-rostral progression (Fisher’s test, *p* value = 0.00001861) or with no LRP (Fisher’s test, *p* value = 0.000006906). There was no difference in severe Braak NFT stage (Fisher’s test, *p* value = 0.2306) or high CERAD score (Fisher’s test, *p* value = 0.7622) between the caudo-rostral pattern group (*n* = 83) and individuals with no LRP (*n* = 180).

Of the 119 subjects with neuropathological AD defined by modified NIA-RI criteria [36] 60 (50%) showed LRP pathology. Of those 36 (60%) exhibited amygdala-based progression pattern, and 24 (40%) caudo-rostral progression pattern. When counted from 119 subjects with neuropathological AD, 30% had amygdala-based and 20% caudo-rostral progression pattern of LRP.

### *APOE* ε4 and LRP progression patterns

*APOE* ε4 was associated with the amygdala-based progression pattern, when comparing with the caudo-rostral progression pattern (Fisher’s test, *p* = 0.001843) or individuals with no LRP (Fisher’s test, *p* = 0.00004611, data shown in Table 3). No significant association with *APOE* ε4 was found, when the caudo-rostral pattern and individuals with no LRP were compared (Fisher’s test, *p* = 0.4457).

## Discussion

Despite the fact that DLB was discovered as a disease entity already 35 years ago, many questions still remain to be solved concerning its pathology, genetics, and clinical characteristics. Common challenges in DLB research include unclear overlap between DLB, AD and PDD, heterogeneity of DLB genetic risk factors, and a shortage of population-based studies, which can assess neuropathology and genetics in a population at large [12, 16, 20, 26, 45, 46]. To address some of these challenges, we investigated LRP in a population-based setting using a wide distribution of tissue samples from spinal cord to neocortical areas without any hierarchical selections. In addition to the anatomical distribution, we investigated the progression patterns of LRP and its relation to AD pathology. We provide neuropathological and genetic evidence that two common progression patterns of LRP pathology exist in the very elderly population: the caudo-rostral pattern consistent with the generally accepted concept of LRP progression (67% of subjects with LRP), and the AD-associated amygdala-based pattern (32% of subjects with LRP). LRP with caudo-rostral or amygdala-based progression patterns were found in 27% and 13% of the whole population, respectively, thus these pathologies represent very common neuropathologies in the very elderly population.

### DLB Consortium classification and LRP progression patterns

This study updates our previous analysis of DLB Consortium LRP types in the Vantaa 85+ sample, which was based on hierarchical selection of selected brain areas and the use of a less sensitive and specific α-synuclein antibody [33]. In the present study, we assessed LRP in 11 anatomical sites with a more sensitive antibody [24, 25] but the results of neocortical areas did not change our previous classification of 43 subjects with neocortical type. However, 11 subjects (9%) with minimal pathology in the medulla were regarded as unclassified, when using the DLB Consortium guidelines. Another population-based study (CFAS) reported that a number of subjects could not be classified according to the DLB Consortium guidelines [47]. They found 8% of the subjects to have exclusively neocortical LRP, whereas we did not find any subjects with neocortical LRP without LRP in other brain areas.

When using our guidelines (systemic anatomical scoring) according to progression patterns, only one subject (1%) could not be categorized. This one individual had severe LRP in all examined brain areas, and thus it was not possible to determine the progression pattern of LRP. It is of note that those 11 subjects unclassified according to the DLB Consortium guidelines could be classified based on our progression pattern guidelines (systemic anatomical scoring). We hypothesize that these subjects actually represent individuals in the early stage of caudo-rostral progression pattern.

Nearly all individuals with the brainstem-predominant type according to the DLB Consortium guidelines showed the caudo-rostral progression pattern, as would be expected based on the PD staging by Braak et al. [6]. The limbic and diffuse neocortical LRP types were mixed, particularly the diffuse neocortical LRP type (Table 2). Subjects with diffuse neocortical LRP type have abundant LRP in most brain areas, and thus categorization of the progression type is challenging. As expected, all subjects classified as amygdala-predominant type showed the amygdala-based progression pattern. We hypothesize that the subjects classified as amygdala-predominant type represent individuals in the early stage of the amygdala-based progression pattern. Our cohort consists of very elderly individuals, most of them showing dense and widely distributed LRP load accumulated possibly during a long time period, and this may have enabled us to observe two distinct progression patterns.

### Association of amygdala-based progression with dementia and survival

In our study, 93% of subjects were with amygdala-based LRP pattern whereas 63% of subjects with caudo-rostral pattern were diagnosed with dementia. The mean age at onset of dementia was 3.3 years earlier in the amygdala-based pattern than in the caudo-rostral pattern, possibly reflecting multipathology, i.e., concomitant AD and LR pathologies in the limbic areas. Interestingly, the mean survival after dementia was 2.7 years longer in the amygdala-predominant versus caudo-rostral pattern and, thus the mean age at death did not differ significantly. Previous population-based studies have not investigated possible associations of dementia, survival, and progression patterns.

### Association of the progression types with AD pathology

The strong association between the amygdala-based pattern and AD pathology is in accordance with previous studies [2, 11, 18, 34, 41, 43]. In our sample, any LRP was found in 50% of subjects with neuropathological AD, which is in line with both population and non-population-based studies [2, 18, 21, 38, 43]. Of the subjects with neuropathological AD in this study, 30% showed amygdala-based and 20% caudo-rostral progression pattern. These figures are somewhat different to those found in the previous study by Uchikado et al. [43] focused on cases of AD, where 18% had AD/amygdala Lewy body pathology and a few more (25%) had a pattern resembling caudo-rostral progression. However, both study design and neuropathological classification scheme were different in this study compared to ours, and hence these studies are not directly comparable. Interestingly, in the present study both progression patterns of LRP were common in the diffuse neocortical LRP type; 40% had the amygdala-based and 58% showed the caudo-rostral progression pattern. Whether this could explain some clinical variation of DLB, needs to be investigated in future studies.

### Association of *APOE* ε4 and the amygdala-based progression pattern

The clear dichotomous association of the *APOE* ε4 carrier status with the amygdala-based progression strongly supports the existence of a biologically distinct AD-associated LRP type. *APOE* has been the strongest and the most replicable finding in genetic studies of DLB [4, 7, 17, 40, 42]. However, the associations between DLB and *APOE* ε4 have been weaker than those found between *APOE* ε4 and AD [15, 35, 42]. In our previous study, an association between *APOE* ε4 and neocortical LRP was found but lost its significance when AD pathological variables were included in the multiple regression model [35], indicating no independent association between LRP and *APOE* ε4. In light of the present results, the *APOE* ε4 association in the Vantaa 85+ and in the other data sets reported thus far may be driven by subjects with amygdala-based LRP with concomitant AD pathology. Our population-based data are in somewhat contrast to a recent large study by Dickson et al., based on a material from a referral-based institution [9], where subjects with low AD pathology were divided into groups, of which “diffuse Lewy body disease” (*n* = 33, median Braak NFT stage III, Thal phase 1) was associated significantly with *APOE* ε4, while no association was found in “transitional Lewy body disease” (*n* = 46, median Braak NFT stage II, Thal phase 1). It is noteworthy that the previously reported “pure DLB” of the Vantaa 85+ material [33] (comparable to the “diffuse Lewy body disease” group reported by Dickson et al.) was not associated with *APOE* ε4 (Supplemental Table 3). Moreover, the material of the study by Dickson et al. was referral-based, younger and with a male predominance, while in the Vantaa 85+ there were only 24% males in the caudo-rostral and 12% in the amygdala-based patterns.

### Strengths and limitations of the study

The strengths of the study include its population-based nature and relative genetic and cultural homogeneity of the Finnish population. The specific antibody clone 5G4 was used in this study, because it has been shown to work well even in preserved samples with long fixation times [25]. We used an objective statistical tool, the *K*-means analysis, to identify different patterns of LRP progression. We compared the results of the *K*-means analysis with those of the systematic anatomical scoring and found 90% concordance between the results. On the other hand, our study population was selected by age, and this should be noticed, when comparing our results with other studies. A limitation of the study is the semiquantitative LRP scoring, which is somewhat subjective and may potentially influence the results. There was only limited clinical Parkinsonism data available in our data set (Supplemental Table 5), as the clinical assessment of the very elderly people was challenging, and thus the clinical significance of progression types could not be comprehensively investigated here. Furthermore, we were not able to study olfactory bulb and nucleus basalis of Meynert since they were not included in the original post-mortem sampling protocol. Population-based studies assessing LRP in the olfactory bulb are currently lacking in the literature, but are warranted as they might reveal invaluable insights into the early development of LRP in DLB [8]. In the future, LRP should perhaps be quantified continuously using computational methodology as has been done with tau pathology [31].

## Conclusion

Our population-based data provide strong neuropathological and genetic evidence that two progression patterns of LRP exist in an elderly population: the caudo-rostral pattern consistent with the generally accepted concept of LRP progression and AD- and *APOE* ε4-associated amygdala-based pattern. Our results support the view that AD-associated amygdala-based LRP is a distinct α-synucleinopathy and we show for the first time in a population-based setting that it is common among the very elderly: it was found in 13% of the whole population, in 32% of the subjects with LRP and in 30% of the subjects with neuropathological AD.

## Electronic supplementary material

Below is the link to the electronic supplementary material.

Supplementary material 1 (PDF 105 kb)
