# Supplementary material for: Lewy-related pathology exhibits two anatomically and genetically distinct progression patterns: a population-based study of Finns aged 85+
Source: Acta Neuropathol. 2019 Sep 7;138(5):771–82. doi: 10.1007/s00401-019-02071-3 (PMC6800868; doi:10.1007/s00401-019-02071-3)
Supplement: Supplementary file 1 — Supplementary material 1 (PDF 105 kb) [file 401_2019_2071_MOESM1_ESM.pdf]

Supplemental material

Acta Neuropathologica

Lewy related pathology exhibits two anatomically and genetically distinct progression patterns - a population-based study of Finns aged 85+

Anna Raunio[1]; Karri Kaivola[2]; Jarno Tuimala[3]; Mia Kero[1]; Minna Oinas[4]; Tuomo Polvikoski[5]; Pentti Tienari[2]; Anders Paetau[1]; Liisa Myllykangas[1]

[1] Department of Pathology, University of Helsinki and HUSLAB, Helsinki University Hospital, Helsinki, Finland

[2] Translational Immunology, Research Programs Unit, University of Helsinki and Department of Neurology, Helsinki University Hospital, Helsinki, Finland

[3] Department of Pathology, University of Helsinki, Helsinki, Finland

[4] Department of Neurosurgery and Department of Pathology, University of Helsinki and Helsinki University Hospital, Helsinki, Finland

[5] Institute of Neuroscience, Newcastle University, Newcastle upon Tyne, NE2 4HH, UK

Corresponding author: Liisa Myllykangas, MD, PhD, Department of Pathology, University of Helsinki and HUSLAB, Helsinki University Hospital, Helsinki, Finland, PL21, 00014 University of Helsinki, Finland (E-mail: liisa.myllykangas@helsinki.fi)

Rules for classifying cases unclassifiable according to DLB guidelines to most comparable category

When at least the substantia nigra sample of brainstem regions was positive, the subject was classified into the brainstem category. If more caudal samples from the substantia nigra were positive and neuron loss at the substantia nigra was moderate or severe, the subject was classified into the brainstem category. If pathology was present in the parietal cortex and temporal cortex, and the density was at least 1+ (meaning more than one field of view per sample 1+), the subject was classified into the diffuse neocortical category. If the density of temporal cortex was at least 1+, but only very mild (1-) pathology was present at the frontal or parietal cortex, the subject was classified into the limbic category. Distribution of pathology in the neocortical regions was considered more important than the density of amygdala pathology. When pathology was present at the parietal cortex, but all neocortical regions were only mild (meaning only one field of view and 1-), the subject was considered belonging to the limbic category. Even if the temporal cortex was 1+, but the parietal 1- and amygdala only mild, the subject was classified into the limbic category. If unilaterality between the hippocampal samples was seen, the subject was classified to the most fitting category considering the staining pattern in its entirety.

Supplemental table 1. Demographic details of the whole Vantaa 85+ study population and of the neuropathologically examined subsample, slightly modified version of the table originally published by Oinas et al 2009 [33].

	Vantaa 85+ Study population (N=565)	Neuropathologic subpopulation (N=304)
Demographic details		
Sex (n, %)		
Men	118 (21)	52 (17)
Women	447 (79)	252 (83)
Age at death (mean ± SD)	91.9 (± 3.6)	92.4 (± 3.7)
Age at death (n, %)		
85-89	188 (33)	82 (27)
90-94	267 (47)	146 (48)
≥ 95	110 (19)	76 (25)
Clinical characteristics		
Dementia status (n, %)		
Dementia	326 (58)	196 (64)
No dementia	239 (42)	108 (36)
Frequency of dementia (n, %)		
Men	63 (53)	30 (58)
Women	263 (59)	166 (66)
Age at onset (mean ± SD)	86.8 (± 4.5)	87.2 (±4.5)
Duration of dementia (mean ± SD)	5.2 (± 3.5)	5.4 (± 3.7)

The study population includes all subjects who deceased during the ten-year follow-up time and had approved to participate in the study.

Supplemental Table 2. Central nervous system (CNS) regions investigated immunohistochemically using α-synuclein antibody (clone 5G4)

CNS Region	Sites investigated within region
Spinal cord	Thoracic (th3-4) intermediolateral horn of the thoracic spinal cord
Spinal cord	Sacral (s1-2) posterior root entry, sacral anterior horn, central canal adjacent to sacral spinal cord
Medulla	Dorsal motor nucleus of vagus IX/X (dm)
Pons	Locus coeruleus (lc)
Midbrain	Substantia nigra (sn)
Basal forebrain	Amygdala
Hippocampus	CA2 and transentorhinal cortex
Cingulate gyrus	Grey matter
Temporal cortex	Grey matter
Frontal cortex	Grey matter
Parietal cortex	Grey matter

Semiquantitative scores: 0=none, 1=mild, 2=moderate, 3=severe, 4=very severe

Supplemental table 3. DLB Consortium classification high likelihood DLB/ neuropathological "pure" DLB previously described by Oinas et al. 2009 [33] divided by the carrier status of *APOE* ε4.

	Pure DLB (Oinas et al.) n=39	Pure DLB *APOE* ε4 yes n=13	Pure DLB *APOE* ε4 no n=24
Caudo-rostral all n=34	34	10	22
Amygdala-based all n=5	5	3	2

Pure DLB: Braak 0-II and LRP limbic or neocortical OR Braak III-IV and neocortical LRP. *APOE* ε4 data not available for two subjects.

Supplemental table 4. Mean values (SD) of Figure 1.

	All			Caudo-rostral			Amygdala-based		
	N	mean	SD	n	mean	SD	N	mean	SD
Spinal S	123	1.60	1.418	82	1.95	1.456	40	0.82	0.931
Spinal Th	124	1.99	1.528	83	2.42	1.499	40	1.05	1.108
Medulla	124	2.48	1.284	83	2.72	1.252	40	1.92	1.185
Pons	124	2.27	1.461	83	2.49	1.476	40	1.78	1.310
Sn	124	2.14	1.309	83	2.14	1.317	40	2.07	1.289
Amy	124	2.41	1.373	83	1.96	1.401	40	3.30	0.723
Ca2	124	1.43	1.263	83	1.19	1.098	40	1.85	1.424
Tox	124	1.78	1.446	83	1.49	1.365	40	2.33	1.439
Cing	124	1.48	1.310	83	1.39	1.248	40	1.62	1.390
Temp	124	1.23	1.268	83	1.00	1.115	40	1.62	1.409
Front	124	0.84	1.007	83	0.78	0.925	40	0.87	1.067
Pariet	124	0.58	0.947	83	0.51	0.771	40	0.65	1.122

Supplemental table 5. LRP progression patterns were compared with clinical parkinsonism symptoms (rigidity and hypokinesia) and substantia nigra neuronal loss (SN neuron loss), originally reported by Oinas et al 2009 [33]

		No LRP n=180	Caudo-rostral n=83	Amygdala-based n=40
Rigidity n=31	no	12	6	3
	yes	6	1	3
Hypokinesia n=32	no	10	6	3
	yes	9	1	3
SN neuron loss n=303	none	6	0	1
	mild	115	36	10
	moderate	54	36 (*)	25
	severe	4	11 **	4

SN neuron loss:
(*) Caudo-rostral LRP progression pattern compared with no LRP (moderate SN neuron loss vs none p=0.0805)
** Caudo-rostral LRP progression pattern compared with no LRP (severe SN neuron loss vs none p=0.0039)
